# Correction: Genetic dissection of Down syndrome-associated congenital heart defects using a new mouse mapping panel

**DOI:** 10.7554/eLife.61021

**Published:** 2020-07-21

**Authors:** Eva Lana-Elola, Sheona Watson-Scales, Amy Slender, Dorota Gibbins, Alexandrine Martineau, Charlotte Douglas, Timothy Mohun, Elizabeth MC Fisher, Victor LJ Tybulewicz

Lana-Elola E, Watson-Scales S, Slender A, Gibbins D, Martineau A, Douglas C, Mohun T, Fisher EMC, Tybulewicz VLJ. 2020. Genetic dissection of Down syndrome-associated congenital heart defects using a new mouse mapping panel. *eLife* **5**:e11614. doi: 10.7554/eLife.11614.

Published 14, January 2016

Recently, a collaborator noticed an error in Table 2. In the column “Dp1Tyb”, the “Total number of CHD” is 24, but the Table only displayed 22 hearts with specific types of defects. This was caused by a typographical error in which 2 hearts with defects had been omitted from the detailed list. One missing sample had a “pVSD + AVSD” defect and the other had “AVSD”. Now with the whole dataset the “Total number of CHD” in Dp1Tyb embryos correctly adds up to 24, as previously indicated.

While re-checking the data for this Table we noticed that in the column “Dp5Tyb” one of the two hearts classified under “pVSD + AVSD” also had an outflow tract defect. Again, this was caused by a typographical error when the Table was compiled. The correction does not affect the number (3) of Dp5Tyb embryos that had CHD.

In addition, for consistency, in the final row, labelled “% of CHD”, we now display numbers with one decimal place.

Table 2 has been corrected accordingly. The corrections do not change any of the graphs based on these data or any of the conclusions that we drew from these data. We apologize for any confusion this oversight may have caused.

